# Congenital anomalies prevalence in Addis Ababa and the Amhara region, Ethiopia: a descriptive cross-sectional study

**DOI:** 10.1186/s12887-019-1596-2

**Published:** 2019-07-11

**Authors:** Molla Taye, Mekbeb Afework, Wondwossen Fantaye, Ermias Diro, Alemayehu Worku

**Affiliations:** 1 Department of Anatomy, School of Medicine, College of Medicine and Health Sciences, the University of Gondar, P.O. Box: 196, Central Gondar, Ethiopia; 2 Department of Anatomy, School of Medicine, College of Health Sciences, Addis Ababa University, Addis Ababa, Ethiopia; 3 School of Dentistry, College of Health Sciences, Addis Ababa University, Addis Ababa, Ethiopia; 4 Department of Internal Medicine, School of Medicine, College of Medicine and Health Sciences, the University of Gondar, Central Gondar, Ethiopia; 5 School of Public Health, College of Health Sciences, Addis Ababa University, Addis Ababa, Ethiopia

**Keywords:** Congenital anomaly, Children, Ethiopia

## Abstract

**Background:**

During the first three months of pregnancy, the developing embryo may be susceptible to external and internal factors, which may lead to structural and functional congenital anomalies. The main objective of this study was to determine the prevalence of congenital anomalies in Addis Ababa and the Amhara region, Ethiopia.

**Methods:**

A descriptive cross-sectional study was conducted on children 0–17 years of age who visited the 16 selected hospitals in Addis Ababa and the Amhara Region between January 1 and July 5, 2015. The proportions of neonates, infants, and children with external and internal congenital anomalies whether the anomalies were major or minor were estimated.

**Results:**

Out of 76,201 children, 1518 of whom 57.6% were male identified with congenital anomalies. The overall proportion of congenital anomaly was 1.99% (95% CI: 1.89–2.091) i.e., 199 per 10,000 children. The proportion of neural tube defects, orofacial clefts, masculo-skeletal system anomalies, syndrome disorders, and cardiovascular system problems were 40.3% 37.7–43, 23.3% 21.3–25.4, 23.1% 20.9–25.2, 8% 6.7–9.4, and 2.6% 1.8–3.4, with a 95% CI, respectively. The majority (72.5%) of the mothers were multigravidae; 38(2.5%) of the mothers and 32(2.1%) of the fathers had history of other children with congenital anomalies. Similarly, 20(1.3%) of the participant children’s mothers and 17(1.1%) of the fathers had familial history of congenital anomaly. Iron folate and multivitamin use by mothers during preconception and early pregnancy was found to be low.

**Conclusion:**

Neural tube defects, orofacial clefts, and musculoskeletal anomalies were the observed prevalent problems. Maternal illness, viral infections, and malnutrition were seen in a significant number of the mothers. Iron folate/folic acid and multivitamin use by the mothers during and before pregnancy was very low.

## Background

During the first three months of pregnancy, the developing embryo may be susceptible to external and internal factors which may lead to congenital anomalies (CAs) [1–3]. CAs which can or cannot be observed at birth but often detected later in life are either structural or functional defects [4–6]. These occur in 3% of all births [7], affecting one in 33 babies [8, 9]; 2–3% [10] of the anomalies which account for 15–30% of the pediatric admissions are detected by the fifth year of life [9].

Genetic and environmental factors as well as maternal health conditions/diseases, substance abuse, and micronutrient deficiencies have linkages with the occurrences of CAs [2, 3, 7, 11–16]; which are also associated with chromosomal abnormalities; occurring due to errors in the numbers or structures of the chromosomes. For many CAs however the causes are still unknown [7, 17–22].

CAs cause deaths of children aged less than 1 year, particularly. Most CAs cause physical and mental disabilities in the affected children [23–26] as well as pregnancy losses through miscarriage and stillbirths [27, 28]. Worldwide, 6% (8.1 million) of new born children have CAs of genetic or partially genetic origins. Moreover, 3.3 million neonates die each year before they reach their fifth year, while 3.2 million children are disabled due to CAs [7, 23, 27–29].

The problems can occur as isolated or multiple anomalies [30] and can affect any part of the organ system of the developing embryo and vary in prevalence from country to country, race to race, or ethnicity to ethnicity [31, 32]. Problems that do not need medical treatment and have either no or just minimal cosmetic effects are known as minor anomalies, while those which require medical treatment [33, 34] and have serious health, psychosocial, and cosmetic effects are called major [35]. About 94% of the major CAs occur in developing countries, and death caused by them accounts for about 95% of all mortalities [23].

CAs can either be visible, like spina bifida (neural tube defects), orofacial clefts, omphalocele, gastroschisis, and reduction limb defects or invisible, such as heart defects, patent ductus arteriousus, tracheoesophageal atresia, and duodenal atresia [36].

In our previously published article, we reported the magnitude of birth defects as 1.9% [37] on the basis of a review of records from 2010 to 2014. The present study was conducted by using primary data collected between January 1 and July 5, 2015 because it was essential to explore the magnitude of the problem by using different data sources. In Ethiopia, CAs are neglected public health problems for parents/families who are affected with the problems. Besides, knowledge on the burden and the etiologies of the anomalies are limited. The main objective of this study was therefore to investigate the situation of CAs in Addis Ababa and the Amhara region and provide valuable information that may be used as baseline for further studies on the problem.

## Methods

### Study sites

Addis Ababa, the capital of Ethiopia and the Amhara region, the second largest in Ethiopia, were purposively selected.

Addis Ababa has an estimated total population of 3,273,001(47.4% male, 52.6% female) [38]. Almost all of the hospitals in the city provide various inpatient and outpatient services, including delivery. Only Cure International Children’s Hospital does not provide delivery services.

The Amhara region has an estimated total population of 20,399,004 (50.1% male, 49.9% female) [38]. The majority of the people live in rural areas. All of the hospitals in the regional state render different kinds of inpatient and outpatient services, including delivery.

### Design of the study

The study was descriptive cross sectional. All children 0–17 years who visited the study hospitals for various medical services were screened for CAs. After diagnosis by pediatricians and other experienced medical doctors, the mothers/caretakers of children with CAs were interviewed to estimate the proportion of victims.

### Sampling and sample size determination methods

Ten hospitals in Addis Ababa (6 public, 4 private) and six in the Amhara region (3 public, 3 private) were purposively included on the basis of case load. To screen children with CAs, we used the single population proportion formula and determined the minimum sample size required. We were bound to consider the prevalence of 1.9% [37] CAs on the 0–17 years of age group reported in Ethiopia. By using a 95% confidence interval, a 0.2% margin of error, and a design effect of 2, the minimal calculated sample size for screening for CAs was 36,950. Then, by adding a 10% contingency, the final sample size required for screening CAs was 41,056. However, to increase the degree of precision of the estimate, all children (76,201) visiting the study hospitals in the two regions during the study were screened for CAs. Then, only the mothers/caretakers of 1518 children with CAs were interviewed. All children with external and internal CAs whether the problems were major or minor were included without considering ethnicity, religion, social strata, residence, region, occupation, and education of parents/families/caretakers. Mothers/caretakers voluntarily participated on behalf of children.

### Study population

The source population was all children in the age group of 0–17 years who visited the health facilities for various medical reasons during the study, while the study population included children 0-17 years who visited the facilities with CAs.

### Data collection method

The data were collected in public and private hospitals which neonates, infants, and children visited for various medical care services. In the two study sites, children with CAs were carefully examined and diagnosed by pediatricians and other experienced medical doctors. After the diagnoses, relevant information was gathered by trained nurses, midwives, other health professionals, and the primary investigator by interviewing only mothers/caretakers whose children had CAs by using the structured questionnaire and a checklist. Data collected on the day the children were diagnosed with CAs included parental socio-economic status, residence, ethnicity, occupation, educational and nutritional status, exposures to teratogenic agents, CA history, pregnancy adverse outcomes, and parity. The data were collected in delivery rooms/wards, neonatal units, pediatric wards, clinics, and centers of cleft lip and palate between January 1 and July 5, 2015.

### Data management and analysis

The data carefully checked daily by collectors were further examined by the primary investigator and entered into Epi-Info version 3.5.1 and transferred into SPSS version 21 for analysis. Data cleaning, error checking, and analysis were conducted by using SPSS, version 21. The confidence interval for the overall proportion of anomalies was calculated manually and the rest of the proportions and statistical analysis were conducted by using SPSS version 21. Descriptive frequency and proportion (with its corresponding 95% CI) were used to describe the results.

### Ethical considerations

The ethical approval letter was collected from Addis Ababa University, College of Health Sciences Institutional Review Board; the National Research Ethics Review Committee; HARI-ALERT Ethical Review Committee; Addis Ababa City Administration Health Bureau Ethical Clearance Committee; and the Amhara National Regional State Health Bureau Regional Health Research Laboratory Center. Support letters were written to zonal health departments and the study hospitals by the health bureaus. The ethical and support letters were submitted to all study hospital administrators/medical directors. The purpose of the study was explained to the participant children’s mothers/caretakers. Data were collected after permission was obtained from the authorities and written consent was obtained from the children’s mother/caretakers. Data collected from the participants were kept in a secured and locked cabinet to maintain confidentiality.

## Results

A total of 76,201 children visited the study hospitals for various medical care and treatment between January 1 and July 5, 2015. Out of these, 1518 were diagnosed with CAs. The overall proportion of CA was 1.99% (95% CI: 1.89–2.09) i.e. 199 children with CAs per 10,000. Of the total 1518 children with anomalies, 57.6% were male and 42.4% female with the age range of 0-17 years. One thousand two hundred eighty-nine (84.9%) of the children were 0-4 years old. About 52.4% were urban and 47.6% rural dwellers. Of the anomalies, the prevalence of neural tube, orofacial clefts, musculoskeletal system, syndrome disorders, cardiovascular system, genitourinary system, ear-nose-eye and head, and other defects were 40.3% (95% CI: 37.7–43), 23.3% (95% CI: 21.3–25.4), 23.1% (95% CI: 20.9–25.2), 8% (95% CI: 6.7–9.4), 2.6% (95% CI: 1.8–3.4), 1.7% (95% CI: 1.1–2.4), 0.7% (95% CI: 0.3–1.2), and 0.2% (95% CI: 0.0–0.5), respectively. The mean age of mothers 28.31 ranged from 15 to 49 years. About 83% of mothers was between 20 and 35 years old (see Table 1). Among the participant mothers, 90.3% were married, 40.4% had no formal education, and 57% were house wives (see Table 1). More than half of the participants were Christians (1043(68.7%)), followed by Muslims (466(30.7%)), and others were only 9(0.6%). The majority, 1470(48.42%) of the participants were Amhara, and Oromo, Guragie, Somali, Tigre, Siltie, and other ethnic groups constituted 870(28.66%), 181(5.96%), 133(4.38%), 122(4.02%), 87(2.86%), 173(5.7%) of the participants, respectively.

**Table 1:** Socio-demographic characteristics of participants, Addis Ababa and the Amhara Region, Ethiopia, 2016

Variable	Addis Ababa(*n* = 1076)		Amhara Region(*n* = 442)		Total	
	Number	(%)	Number	(%)	Number	(%)
Child Gender						
Male	609	56.6	265	60	874	57.6
Female	467	43.4	177	40	644	42.4
Child Age						
0-4 year	909	84.5	380	86	1289	84.9
5-9 year	109	10.1	50	11.3	159	10.5
10-14 year	52	4.8	11	2.5	63	4.2
13-17 year	6	0.6	1	0.2	7	0.5
Residence						
Urban	628	58.4	167	37.8	795	52.4
Rural	448	41.6	275	62.2	723	47.6
Children mother age						
< 20 years	60	5.6	16	3.6	76	5
20-35 years	937	87.1	323	73.1	1260	83
> 35 years	79	7.3	103	23.3	182	12
Marital status of mothers						
Married	973	90.4	397	89.8	1370	90.3
Never married	58	5.4	29	6.6	87	5.7
Divorced/widowed	45	4.2	16	3.7	61	4
Occupation(child mother)						
Housewife	611	56.8	249	56.3	860	56.65
Farmer	103	9.6	120	27.1	223	14.69
Employee	208	19.3	27	6.1	235	15.48
Merchant	51	4.7	24	5.4	75	4.94
Other^a^	103	15.8	22	5	125	8.23
Occupation(child father)						
Employee	469	43.6	92	20.8	560	36.89
Farmer	333	30.9	276	62.4	609	40.12
Jobless	158	14.7	52	11.8	210	13.83
Student	93	8.6	17	3.9	110	7.25
Other^a^	23	2.1	5	1.1	28	1.84
Wealth quantile						
Low income	467	43.4	179	40.5	646	42.55
Middle income	521	48.4	223	50.5	744	49.01
High income	88	8.2	40	9.0	128	8.43
Educational level						
No education	366	34	248	56.1	614	40.4
Primary school(1-8th grade)	348	32.3	119	26	467	30.8
Secondary school(9-12grade)	251	23.3	53	12.0	304	20.02
College and above	111	10.4	22	5	133	8.76

Others^a^ = Somali, Afar, Guragie, Argoba Benishangul, merchant, jobless, student, nomad

About 38(2.5%) of the mothers and 32(2.1%) of the fathers had history of other children with CA. Similarly, 20(1.3%) of the participant’s mothers and 17(1.1%) of the fathers had familial history of CA.

The majority, (72.5%), of the mothers were multigravidae. That is, CAs were more frequent in multigravidae and lower in primigravidae mothers. Some, (10.6%), of the mothers had history of 1–2 preterm babies, 11% 1–2 miscarriages, and 6.1% 1–2 still births. Of the children, 34.15% were 2nd order; 17.7% of the mothers had no antenatal care (ANC) follow ups, while 41.1% started ANC visits at 4-6 months (see Table 2).

**Table 2:** Selected reproductive history of children’s mothers, Addis Ababa and the Amhara Region, Ethiopia, 2016 (*n* = 1518)

Characteristics		Frequency	Percent
Gravidity/Parity	Prim gravid	417	27.50
	Multi-gravid	1101	72.50
Total number of pregnancy	1	417	27.50
	2–3	627	41.30
	4–5	316	20.80
	> 5	158	10.40
Full and post term	Post term	38	2.50
	1–2 full term	842	55.50
	3–4 full term	412	27.10
	> 4 full term	226	14.90
Preterm	None	1357	89.40
	1–2	161	10.60
Elective termination	None	1427	94.00
	1–2	91	6.00
Spontaneous miscarriage	None	1350	88.90
	1–2	167	11.00
	3–4	1	0.10
Still birth	None	1425	93.90
	1–2	93	6.10
Living children	None	35	2.30
	1–2	872	57.40
	3–4	410	27.00
	> 4	201	13.20
Infant/child death	None	1358	89.5
	1–2	152	10
	3–4	7	0.5
	> 4	1	0.1
Birth Order in multigravidae	1st	77	6.99
	2nd	376	34.15
	3rd	253	22.98
	4th	159	14.44
	5th	113	10.26
	>5th	123	11.17
Antenatal Care (ANC)	Yes	1249	82.3
	No	269	17.7
Gestational age at first ANC	None	269	17.7
	1-3 months	625	41.2
	4-6 months	624	41.1

**Fig. 1:**
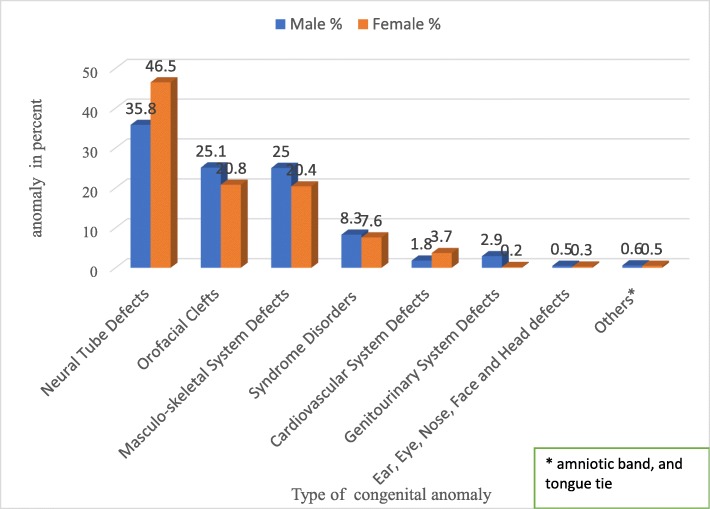
Distribution of congenital anomalies by sex, Addis Ababa and the Amhara Region, Ethiopia, 2016

As presented in Table 3, the highest proportion (40.3%) of the anomalies were neural tube defects. With regard to sex, about 46.5% females had neural tube defects, whereas 25.1% of the males had orofacial clefts. Likewise, 25.0% of the males had masculo-skeletal anomalies, while 3.7% of the females had cardiovascular anomalies. On the other hand, there were 7.6% syndrome disorders (Down, Crouzon, Edward, TAR, and Rubellar) among the female children. Almost 2.9% of the genitourinary system anomalies occurred in the males (see Fig. 1). The distributions of some selected anomalies by type are presented in Table 4.

**Table 3:** Congenital anomalies prevalence by study site and type of anomaly, Addis Ababa and the Amhara Region, Ethiopia, 2016

Variable	Addis Ababa		Amhara Region		Total	
	Number	(%)	Number	(%)	Number	(%)
Neural Tube Defect	478	44.4	134	30.3	612	40.3
Orofacial Cleft	210	19.5	144	32.6	354	23.3
Musculoskeletal System Defects	290	27.0	60	13.6	350	23.05
Syndrome Disorder	29	2.7	93	21.0	122	8.04
Cardiovascular System defects	38	3.5	2	0.5	40	2.6
Genitourinary System Defects	22	2.0	4	0.9	26	1.7
Ear, Eye, Nose, Face and Head Defects	4	0.37	2	0.45	6	0.39
Others^a^	5	0.46	3	0.67	8	0.53

^a^amniotic band, tongue tie

**Table 4:** Percentage distribution of selected congenital anomalies by types of anomaly, Addis Ababa and the Amhara Region, Ethiopia, 2016

Type of congenital anomalies	Frequency	Percent
Spina bifida	268	17.70
Hydrocephalus	180	11.90
Spina bifida with hydrocephalus and club foot	58	3.82
Anencephaly	36	2.40
Bilateral cleft lip and palate	25	1.60
Bilateral cleft lip	24	1.60
Unilateral cleft lip and palate	13	0.90
Ventricular septal defect	20	1.30
Omphalocele	14	0.90
Syndrome disorders (Down syndrome, Edward Syndrome etc)	103	6.80
Bilateral club foot	108	7.10
Unilateral club foot	72	4.74
Imperforated anus	23	1.50
Spine defects(Congenital Scoliosis)	51	3.30
Hypospadia	15	1.00
Tracheoesophageal fistula	10	0.70

As shown in Table 5, among the children’s mothers, about 9.7% had maternal illness; 17.1% had history of passive smoking of cigarettes; 11.5% used un-prescribed/illicit drugs; 5.5% had history of blood relationship with the children’s fathers; 4.9% had history of exposure to chemicals; and 1.8% had history of exposure to radiation during early pregnancy.

**Table 5:** Response of children’s mothers (“yes” response) for selected risk factors for congenital anomalies, Addis Ababa and the Amhara region, Ethiopia, 2016 (*n* = 1518)

Characteristics	Frequency	Percent
Maternal illness during early pregnancy	148	9.7
Smoked cigarette during pregnancy	13	0.85
Passively Smoked during pregnancy	259	17.1
Drunk alcohol during pregnancy	403	26.5
Used un-prescribed/illicit drug early pregnancy	175	11.5
Exposure to radiation (child mother)	28	1.8
Child father exposed to radiation	29	1.9
Blood relation between child parents	83	5.5
Exposed to chemicals	74	4.9
Other exposure that concern parents	86	5.7
Viral infection during early pregnancy	14	0.9
Contraceptive pills use around conception	154	10.1
Anemia during early pregnancy	244	16.1
Fertility enhancing drug use	38	2.5
Folic acid use during pregnancy	75	4.94
Iron use during early pregnancy	53	3.5
Multivitamins use before pregnancy	13	0.9
Multivitamins use during pregnancy	16	1.1
Food shortage during pregnancy	160	10.5
Used vegetables/fruits during pregnancy	458	30.2

Out of the mothers, 0.9% had viral infections and 16.1% anemia during the first 3 months of pregnancy. Around 4.94% of the mothers took folic acid during early pregnancy, while the majority of the mothers (95.06%) took (consumed) no folic acid during early pregnancy. Besides, 3.5% of the mothers took iron for the treatment of anemia (see Table 5).

Almost 10.5% of the mothers had food shortage during pregnancy. In terms of vegetables/fruits use, nearly 30.2% of the mothers took vegetable/fruit combined with other food items (staple food) during pregnancy, while 69.8% took (consumed) no vegetables/fruits throughout their pregnancy. Only 0.9 and 1.1% of the mothers consumed multivitamins before and during pregnancy, respectively (see Table 5).

The proportion of anomalies were 979(64.5%) for Addis Ababa and 539(35.5%) for the Amhara region. The frequency of CAs by study hospitals in Addis Ababa and the Amhara region are presented in Table 6.

**Table 6:** Congenital anomalies prevalence among the study hospitals, Addis Ababa and the Amhara Region, Ethiopia, 2016

Hospital name, level by national millennium standard, ownership, and study place			
	Level of hospital	Number of CA cases	Percent
Addis Ababa			
Public Hospitals			
Zewditu Memorial Hospital	Secondary	495	46.0
Tikur Anbesa General Specialized Hospital	Tertiary	242	22.5
Yekatit 12 Hospital Medical College	Secondary	81	7.5
St. Paul’s Hospital Millennium Medical College	Tertiary	25	2.3
ALERT Center (Hospital)	Secondary	12	1.1
Ghandi Hospital	Secondary	7	0.7
Private Hospitals			
Cure Ethiopia International Children’s Hospital	^a^Special	171	15.9
Addis Hiwet General Hospital	Secondary	12	1.1
MCM Korean General Hospital	Secondary	20	1.9
Betsegah Special Women’s and Children’s Hospital	^a^Special	11	1.0
Amhara Region			
Public Hospitals			
Desse Referral Hospital	Secondary	160	36.3
Felegehiwet Comprehensive Specialized Hospital	Tertiary	78	17.6
University of Gondar Specialized Hospital	Tertiary	78	17.6
Private Hospitals			
Gamby Teaching Medical Sciences College Hospital	Secondary	86	19.5
Ibex General Hospital	^a^Secondary during data collection period, but now leveled as primary	24	5.4
Selam General Hospital	Secondary	16	3.6

^a^*CA* Congenital Anomaly

One thousand three hundred seventy-nine (90.8%) of the anomalies were single, while 139(9.2%) were multiple (i.e. more than one anomaly in one child in two or more organ systems). About 39(2.6%) of the victims of neural tube defects had other associated CAs (i.e. multiple anomalies). Other anomalies that had associated CAs, just like neural tube defects were Down syndrome and omphalocele, which accounted for 7(0.46%) and 5(0.33%) of the participants, respectively. Furthermore, 1499(98.7%) of the anomalies were major, whereas 19(1.3%) were minor.

## Discussion

In this study, the overall proportion of CAs was 1.99%, almost 2%. This finding is quite close to the result (1.9%) of our previous study on birth defects [37]. Another point to consider here is that our work published previously was carried out by using record reviews from 2010 to 2014. In contrast, the present study was conducted by using primary data (by interviewing children’s mothers/caretakers). The similarity of the two findings may be due to the correspondence of the participants in terms of some characteristics, for instance, residence, geographical area, socioeconomic status, and behavioral factors. Here, the point is to check whether it is necessary to determine the magnitude of CAs by using a variety of data sources at different time or in various years. Thus, these studies could help to find out the similarities or differences in magnitude of two different data sources.

Furthermore, the finding of this study is similar to that of a study conducted in Egypt (2%) by Shawky and Sadik [33], and close to the result of a study done in India (2.22%) by Sarkar et al. [39], but higher than those of studies carried out in Lahore (0.75/10,000) by Parker et al. [40], and Saudi Arabia (1.14%) by Al Bu Ali et al. [41], and less than the findings of studies conducted in Lebanon (2.4%) by Francine et al. [7], and Iran (2.8%) by Karbasi et al. [16]. Again, the finding of the present study is lower compared to those of studies conducted in Tanzania (29%) [42], Nigeria (28.15 per 1000 children) [43], and Palestine (21%) [29], and Gaza (14%) [44]. Furthermore, the finding of this study was less than the findings of those studies conducted in the United States of America, the United Kingdom, Italy, and Canada [45–48]. These differences could be due to variations in environmental factors and the instruments used for diagnosing CAs, such as ultrasound, x-ray, electrocardiograph, and magnetic resonance imaging (MRI scan). In this study, we didn’t use the magnetic resonance imaging device because the majority of the study hospitals had no such devices during the data collection. Basically, we are confident that the finding of our study is adequate to give a clear picture of the situation of CAs in Ethiopian. In addition, from the present study, we can learn that CAs are a big problem that merit due attention. Also, it should be noted that Ethiopia has no surveillance and registry systems for CAs which are lifelong risks for citizens. Hence, policy makers, programmers, and health care providers should plan to teach the community about CAs and schedule for surveillance and registry systems, and monitor the occurrence of the problem in the population and provide comprehensive treatment, care, and rehabilitation services for affected children.

In the current study, the most frequent CAs were neural tube defects. This high rate could be due to no or low use of iron folate during peri-conception and early pregnancy. Moreover, the high prevalence of neural tube defects could be due to the fact that children with neural tube defects survive despite the dangerous nature of the problem. Studies conducted in Tanzania [42], Nigeria [43], Palestine [29], and China [49] reported the occurrence of central nervous system anomalies as 29.8, 6.87, 18.81 and 20.1%, respectively. These findings suggest that central nervous system anomalies are the most frequently manifested problems.

In the present study, spina bifida were the most frequent neural tube defects, followed by hydrocephalus, and anencephaly. These findings were different from the spina bifida and anencephaly of 10.6 and 6.9 per 10,000, respectively, reported in China [49]. This variation may be due to genetic/environmental factors and low use of iron folate.

In this study, the second most frequently observed CAs were orofacial clefts followed by masculo-skeletal system anomalies, syndrome disorders (Down, Crouzon, Edward, and congenital Rubellar) plus cardiovascular and genitourinary system anomalies, and others. However, this finding does not correspond to those of other studies in terms of proportion and sequence.

In our study, more males were affected than females by most types of the CAs observed. The reason why we showed the difference between sexes was to indicate the proportion of anomalies among male and female children. Similar findings, which showed that more males were affected than females reported the contrast as follows: Nigeria 52 and 48% [43], Iran 51.9 and 48.1% [10], and Pakistan 68.3 and 31.7% [16]. The reason why males were more affected by CAs than females may be that chromosomal abnormalities and mutations in genes that influence the genetic pathways may be more common in males than in females. This, however needs further scientific investigations.

Furthermore, in the present study, the majority of the anomalies were major rather than minor. This could be due to the fact that children with minor anomalies were not visiting hospitals for diagnosis. Likewise, the majority of the anomalies were single, while the minority were multiple. Multiple anomalies were observed in children with neural tube defects. Similarly, children who had syndrome disorders (for example Down’s syndrome) and omphalocele were seen with other associated CAs. This finding was different from those of other studies maybe due to differences in causative agents, study places, and genetic variations.

In the present study, the majority of the mothers were multigravidae rather than primigravidae, showing the positive relationship between gravidity and CAs. Similarly, Sarkar et al. [39], Lei et al. [50], and Anyanwu et al. [43] reported associations between gravidity and CAs.

Some of the mothers reported that they had maternal illness; some said they were passive smokers; others informed us that they suffered from viral infections; and a small group reported that they were malnourished during early pregnancy. On the other hand, other similar studies indicated that malnutrition, smoking cigarettes, and illicit drugs were associated with CAs [51]. However, it should be noted that cigarette smoking is not common among Ethiopian women.

In our study, folic acid/iron folate, vegetable/fruit use, and multivitamin consumption before and during pregnancy were very low. This finding was similar with our previous study [52]. This might be due to the fact that the majority of the women could not get iron folate/folic acid and multivitamin in their peri-conception and early pregnancy. In addition, it might be due to low awareness of mothers about iron folate, multivitamin, and vegetable uses for normal body functions and healthy living. Several studies suggested that folic acid [53–55] and multivitamin containing folic acid usage during early pregnancy reduce/protect from the occurrence of CAs [55–61].

In this study, parental lifestyle factors, such as alcohol use, un-prescribed/illicit drugs, blood relationship among parents, exposure to chemicals, exposure to radiation, preterm, miscarriage, still birth, infant death, family history, birth order, anemia, and occupation had notable relationships with CAs. Furthermore, many studies indicated that the associations of the factors mentioned and CAs were positively related [10, 13, 36, 49, 62–66].

The limitation of this study was that it was not supported by special tests, such as genetic/chromosomal defect laboratory investigations and viral infection markers. In addition, medical geneticists and pediatric surgeons were not involved in the study. The other inherent limitation was recall bias. To minimize the bias, we made efforts to retrieve information by giving enough time to participants to enable them remember events during pregnancy and after delivery. We wish to suggest that large population-based studies are required to determine the prevalence of CAs rather than hospital-based attempts like ours.

## Conclusion

In conclusion, this study indicated that neural tube defects, orofacial clefts, and musculoskeletal anomalies were momentous and influential problems which need attention. Maternal illnesses, viral infections, and malnutrition were observed in a significant number of mothers. In addition, iron folate/folic acid and multivitamin use by mothers was very low. On the whole, the proportions observed in the study indicated that a significant number of children were affected and suffered from the impacts of CAs. The problem thus necessitates immediate preventive actions. One of the prevention methods is iron folate supplementation. Therefore, women in the reproductive age groups should be supplemented with iron folate during peri-conception and early pregnancy by health care providers. Further investigations should also be conducted to explore/identify the associated risk factors of CAs and determine prevalence rates. We recommend that researchers conduct analytical and home-based birth studies in the future.

## Data Availability

The data set supporting this study are available in the manuscript.

## Abbreviations

ANC: Antenatal care
CA: Congenital anomaly
CAs: Congenital anomalies
CI: Confidence interval
